# Reviewed article: Costa AF, Tavares LF, Junior PCPC, Barbosa RBC, Honório OS, Oliveira ACR, Cardoso LO. Adaptation of a pediatric hospital in the city of Niterói, State of Rio de Janeiro, to the recommendations of the Brazilian Dietary Guidelines in 2023. Epidemiol Serv Saude. 2025:34;e20240532

**DOI:** 10.1590/S2237-96222025v34e20240532.c

**Published:** 2025-08-08

**Authors:** Nicole Pischel

**Affiliations:** Universidade de Sao Paulo

**Table:** 

Rating	Excellent	Good	Average	Below average	Poor
Interest			✓		
Quality			✓		
Originality		✓			
Overall		✓			

**Table:** 

Questionnaire	Yes	No	Not applicable
Does the manuscript contain new and significant information to justify publication?	✓		
Does the Abstract (Summary) clearly and accurately describe the content of the article?	✓		
Is the problem significant and concisely stated?	✓		
Are the methods described comprehensively?	✓		
Are the interpretations and conclusions justified by the results?	✓		
Is adequate reference made to other work in the field?	✓		

## Manuscript Structure

Length of article is: Adequate

Number of tables is: Adequate

Number of figures is: Adequate

Please state any conflict(s) of interest that you have in relation to the review of this paper (state “none” if this is not applicable): None

## Comments

Prezado Autor.

Seguem recomendações ao seu manuscrito:

A pergunta de pesquisa precisa ser mais bem definida, com os objetivos mais bem delimitados.

Explique como os vieses foram minimizados e a validade interna foi garantida. Como foi realizada a capacitação.

Os métodos da fase 1 módulo 1 precisam ser descritos com mais detalhes para permitir a replicação do estudo por outros pesquisadores.

No módulo 3, é importante que esse instrumento utilizado seja confiável e validado.

No módulo 4, descreva o instrumento de coleta de dados e avaliação. É válido inserir o questionário utilizado como anexo. Importante realizar a validação e Se o instrumento não foi validado, discuta as limitações dessa falha.

Em relação a fase 2 do seu estudo, não está adequada para esse momento. para que uma Nota Técnica seja realizada, deve ser descrita com mais detalhes, levantamento de concordância entre a equipe de elaboração, entre os usuários do serviço e o serviço. Desse modo, seria interessante que essa fase seja removida do atual manuscrito e posteriormente um novo estudo seja realizado de forma completa, abraçando de fato todas as frentes desse assunto.

Em relação as resultados, o módulo 1 deve ser melhor descrito em seus métodos de como foram medidos e avaliador. Ou então removidos. Importante levantar dados concretos.

Nos resultados do módulo 2, discutir e levantar a limitação do estudo por conta dos fatos ali descritos.

E em relação as resultados no módulo 4, descreva com mais detalhes como essa avaliação foi realizada numericamente.

Novamente, sobre a fase 2 do estudo deve ser realizada de forma mais sistematizada e sua metodologia mais robusta. Recomendo que esse artigo se contente apenas com a fase 1, e um novo estudo seja operado contemplando a Fase 2. Sendo assim, revisar o texto completo adequando ao novo formato.

Fico à disposição para qualquer dúvida.

Atenciosamente. 

